# Confusion and Hallucination: A Case Report of an Unusual Presentation of COVID-19

**DOI:** 10.1155/2021/3794019

**Published:** 2021-12-30

**Authors:** Rawabi Aljumaiah, Wael Alturaiki, Bandar Alosaimi

**Affiliations:** ^1^Department of Internal Medicine, King Fahad Medical City, Riyadh 11525, Saudi Arabia; ^2^Department of Medical Laboratory Sciences, College of Applied Medical Sciences, Majmaah University, Majmaah 11952, Saudi Arabia; ^3^Research Center, King Fahad Medical City, Riyadh 11525, Saudi Arabia

## Abstract

Besides respiratory symptoms, COVID-19 disease has a wide range of clinical, subclinical, and atypical presentations reported previously. Here, we report the case report of a middle-aged man, with no previous known medical illness, who presented with a 5-day-history of anxiety, fever, confusion, and hallucinations. Patient's SARS-CoV-2 polymerase chain reaction test was positive, and he underwent daily vital signs and respiratory, cardiovascular, and abdominal examinations. Chest radiography, electrocardiogram, microbial culture, biochemistry, and toxicology tests were also investigated. In this report, a case of COVID-19 is described with an unusual presentation of confusion and hallucinations in the absence of severe upper respiratory or constitutional symptoms. The earlier recognition of atypical manifestation, the safer the practice, with optimal timely diagnosis, and less anticipated outbreaks in healthcare facilities. Further studies are needed to establish the underlying pathophysiological mechanisms involved.

## 1. Introduction

It has been widely recognized that fever and respiratory symptoms are a typical presentation of the coronavirus disease 2019 (COVID-19) infection, however, considering other atypical presentations is crucial for accurate diagnosis and management of patients and also for awareness of doctors. The World Health Organization (WHO) guidance for public health surveillance of COVID-19 provides criteria for clinicians and healthcare workers to identify suspected, probable, and confirmed cases of severe acute respiratory syndrome coronavirus 2 (SARS-CoV-2) infection [1]. However, atypical signs and symptoms to adequately assess the effects of COVID-19 on brain health were not elaborated. An observational study of more than 230,000 patient health records revealed that one in three COVID-19 survivors received a neurological or psychiatric diagnosis within six months of infection. The major neurological or psychiatric outcomes were intracranial haemorrhage, ischaemic stroke, parkinsonism syndrome, dementia, anxiety disorder, and psychotic disorder [2]. Guillain-Barré syndrome following COVID-19 was also reported within 3 weeks of SARS-CoV-2 infection [3]. Similar to SARS-CoV and Middle East respiratory syndrome corona virus (MERS-CoV), SARS-CoV-2 was reported to cause confusion and delirium in 27.9% of patients during the acute illness [4].

Moreover, it has been reported during SARS-CoV-1 epidemic that there were individuals who have been admitted with atypical symptoms and were undiagnosed with SARS-CoV-1 [5]. A case series of 214 patients in Wuhan found out that severe COVID-19 infection commonly presents with neurological symptoms like acute cerebrovascular accident, consciousness impairment, and skeletal muscle injury compared with nonsevere patients [6]. These scenarios point toward emphasizing the importance of interdisciplinary teamwork, allowing clinicians to collaboratively recognize the real prevalence and clinical significance of any atypical presentations in COVID-19 patients. This also signifies the importance of developing flexible policies with broader capacity to review our screening protocols and consider other atypical symptoms among the criteria beyond respiratory illness [6].

Neurological and psychiatric complications have been reported as postacute COVID-19 side effects and/or long-term neurological or psychiatric complications [7]. However, this report describes an unusual presentation of confusion and hallucinations in the absence of severe upper respiratory or constitutional symptoms during the infection. This report also reflects on the importance of proper neurologic and psychiatric assessment following COVID-19.

## 2. The Case Presentation

A 34-year-old man not known to have any chronic medical illnesses presented to the emergency department (ED) at King Fahad Medical City with a new onset of hallucination and a 5-day history of anxiety. The patient had no history of headache, visual changes, seizures, or body weakness. Patient's symptoms started during the COVID-19 lockdown from May 23 until May 27 in Riyadh, Saudi Arabia. In his country of origin, the patient lost three members of his family because of SARS-CoV-2 infection before his new presentation started. He was initially having restlessness, insomnia, and deep fears of death, which had been noticed by his wife. A psychiatry physician examined him and prescribed 10 mg daily of escitalopram. Five days after starting the new drug, the patient developed hallucinations, visual and auditory, along with anxiety, and aggressive behavior as his wife described. He was brought to our institution on 23-Jun-2020. Upon arrival in the ED, his wife reported symptoms of visual and auditory hallucinations, as he was seeing and talking gibberish, speaking short religious phrases, and hearing voices. He was not consuming alcohol, neither did he smoke, nor abused drugs. The patient underwent laboratory testing on a daily basis throughout the course of hospitalization (Table 1).

On initial examination, the patient was conscious, alert, and oriented to the time and place but not to person. His vital signs at the ER triage revealed an elevated blood pressure (BP) of 150/95 mmHg, a fever with an oral temperature of 38.6°C, a heart rate of 95 beats/minute, a respiratory rate (RR) of 25/minute, and a stable oxygen saturation (SpO2) of 97% on room air. His cranial nerves were intact; there was no neck stiffness nor photophobia, nor any facial asymmetry. A motor examination, sensory, reflexes, cerebellum, and gait were all intact, and the Glasgow Coma Scale (GCS) score to assess his level of consciousness was 13/15 as the verbal response was inappropriate words (level 3 in GCS score) [8]. His flexor plantar response was normal. The findings of respiratory, cardiovascular, and abdominal examinations were unremarkable.

**Figure 1:**
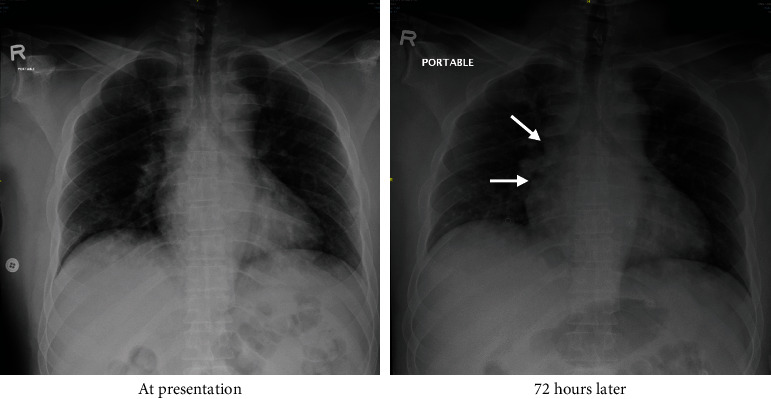
Chest radiograph on the day of onset of symptoms showing no evidence of pneumonia nor any radiological findings of acute respiratory distress syndrome (ARDS). At 72 hours: ground-glass opacification and alveolar consolidation were seen in the middle of right lobe (arrows).

The working diagnosis was acute confessional state. A nasopharyngeal swab for COVID-19 was done in the ED triage giving his recent history of contact with sick patients in his family, and the patient was admitted to isolation room with close monitoring. Lumbar puncture and plain computed tomography (CT) scan of the brain could not be performed because of patient's restlessness and aggressive behavior, which could not be controlled by midazolam. His chest radiograph was unremarkable (Figure 1); electrocardiogram (ECG) showed normal sinus rhythm. The patient was initially managed as a case of delirium and rhabdomyolysis with intravenous (IV) hydration (0.45% normal saline). Cultures from sputum, blood, and urine were requested, and the patient was empirically on piperacillin/tazobactam antibiotics at a dose of 2.25 mg every 6 hours. Toxicology urine screen was sent for amphetamine, and regular fixed doses of antipsychotics (haloperidol) through intramuscular route and benzodiazepines (lorazepam) were prescribed.

Twenty-four hours later, SARS-CoV-2 RT-PCR screening test yielded positive. The patient was afebrile with oral temperature of 37.2°C, SpO2: 98% on room air, BP: 132/80 mmHg, RR: 20/minute, and he was confused with GCS score 13/15 speaking inappropriate words. His aggression and anxiety improved but he was not stable for CT of the brain.

Forty-eight hours after patient's initial presentation, his vital signs were reassuring with oral temperature of 36.6°C, BP: 130/73 mmHg, and SpO2 of 97% on room air, and he was calmer but still confused with GCS score 13/15. He was reassessed, and the regular fixed antipsychotic doses were modified to pro re nata (PRN). Moreover, patient's overall clinical and biochemical status was improving, with supportive care and antibiotics.

Seventy-two-hour postadmission, results from a septic blood screen and urine culture were negative, and patient's vital signs in the morning were stable with a pulse of 90 beats/minute, BP: 135/80 mmHg, SpO2 of 98% on room air, RR: 20/minute, and GCS score: 13/15. He was scheduled for CT brain at evening time; however, his heart rate increased to 110-115 beats/minute around 15:00 pm, ECG showed sinus tachycardia with normal QTc (0.43 s), then gradually, his respiratory rate increased from 20 to 30/minute, and the patient was given bolus of 1 liter IV normal saline. Stat chest radiograph, arterial blood gas, cardiac enzymes, and D-dimer tests were requested. The patient became more tachypneic with RR of 45/minute, dropping GCS score to 10/15, and a high heart rate of 150 beats/minute. The patient collapsed, and cardiopulmonary resuscitation was conducted for 45 min with no response. Death was declared at 18:35 pm.

## 3. Discussion

Since the COVID-19-related respiratory illness presents typically as pneumonia, atypical psychiatric presentations should not be excluded. Indeed, our patient presented with unusual COVID-19 signs and symptoms of confusion and hallucination that did not meet the current WHO case definition criteria. Yet, the United States' Center for disease control and prevention (CDC) recommends that “clinicians should use their judgment to determine if a patient has signs and symptoms compatible with COVID-19 and whether the patient should be tested,” advising that decisions on which patients receive testing should be based on the local epidemiology of COVID-19, as well as the clinical course of illness [9]. Therefore, clinicians should be mindful of the possibility of any atypical presentations, and correlations with new symptoms should be noted carefully.

This case report lay emphasis on the importance of interdisciplinary teamwork, allowing clinicians to collaboratively recognize the real prevalence and clinical significance of any atypical presentations in COVID-19 patients. Narrowing the criteria for COVID-19 testing to only typical symptoms may lead to unreported community spread of the disease and silent transmission of the infection in hospitals [10]. On the other hand, the balance between utilizing resources and infection control is crucial since widespread testing without clear clinical indications is relatively waste of resources. Therefore, it is important to balance two concepts: remedying testing gaps is imperative, yet more testing is not always better [11]. The patient described in this report was early recognized because of interdisciplinary teamwork and protocol flexibility to consider other atypical symptoms among the criteria beyond respiratory illness [6]. This highlights the importance of flexible testing policy, which does not exclude nonspecific signs and symptoms of COVID-19 in form of neurological and psychological manifestations.

In conclusion, as the coronavirus-related respiratory illness presents clinically as pneumonia, the insight we are reporting here is that clinical presentations may not always be a typically pneumonia-like illness, we rather have observed confusion and hallucinations as atypical presentation of COVID-19. Furthermore, neurological and psychiatric complications have been reported as postacute COVID-19 side effects and/or long-term complications [7], yet, our findings highlight the importance of considering confusion and hallucinations as atypical presentations during the infection of COVID-19. The recognition of this atypical presentation, utilization of a more flexible screening strategy, and early interdisciplinary medical care intervention are crucial measures for infection control. We believe that our finding may become a hypothesis generator for further studies investigating the prevalence and clinical significance of confusion and hallucinations in COVID-19 patients and any underlying pathophysiological involved mechanisms.

## Figures and Tables

**Table 1:** Diagnostic results throughout the course of hospitalization.

Diagnostic test	Panel	At presentation	24 hours	48 hours	72 hours	Normal ranges
Blood work up	White blood corpuscles	10.81	12.2	8.58	9.37	3.90-11.00 10^3^/*μ*L
	Hemoglobin	16.9	15.0	15.1	15.1	11-16g/dL
	Platelet	247	265	230	171	100-450 10^3^/*μ*L
	Neutrophils ×109/L	7.6	8.08	5.61	6.65	1.35-7.50
	Lymphocytes ×109/L	2.29	3.21	2.26	1.83	1.50-4.30
	Monocytes ×109/L	0.83	0.92	0.65	0.83	0.25-1.0

Thrombosis marker	D-dimer *μ*g/mL	2.94	2.95	4.86	42.44	<0.5

Electrolytes and renal profile	Sodium, mmol/L	148	150	144	146	136-145
	Potassium, mmol/L	3.33	3.63	4.26	3.62	3.40-4.40
	Bicarbonate, mmol/L	17.4	22.3	21.0	25.6	22-29
	Chloride, mmol/L	111	111	117	109	98.00-107
	Corrected calcium, mmol/L	n/a	2.28	2.22	2.19	2.10-2.55
	Magnesium, mmol/L	n/a	1.06	N/a	n/a	0.66-1.07
	Phosphorus, mmol/L	n/a	1.26	1.29	1.13	0.74-1.52
	Urea, mmol/L	9.3	13.6	12.3	5.8	2.50-6.70
	Creatinine, unit/L	116	94	101	81	49.00-90.00
	Creatine kinase, unit/L	6672	8300	4730	1975	

Cardiac enzyme	High-sensitivity troponin, ng/L	15.9	13.0	14.2	15.0	<4.0
	Brain natriuretic peptide, pg/mL	14.3	n/a	n/a	n/a	<73

Liver function and coagulopathy	Alanine aminotransferase (ALT), unit/L	101	115	111	99	7-56
	Aspartase transaminase (AST), unit/L	134	130	125	99	5-40
	*γ*–Glutamyltransferase (GGT), unit/L	96	70	73	46	10-48
	Bilirubin direct *μ*mol/L	4.4	7.2	8.7	8.0	0-5
	Bilirubin indirect *μ*mol/L	8	9.6	9.1	10.2	0-13
	Albumin g/L	41.9	39.9	36.5	32.1	35-52
	Prothrombin time/s	14.2	15	17.5	13.9	10-13
	Activated partial trombomplastin time (aPTT) time/s	27.5	26	26.3	29.2	25-36
	International normalized ratio (INR)	1.20	1.30	1.47	1.17	<1.1

Inflammatory markers	Lactate, mmol/L	4.21	1.75	1.17	2.43	0.5-2.20
	Ferritin, ng/mL	2420	2408	2142	2132	24 - 336
	C-reactive protein (CRP), mg/L	25.5	n/a	n/a	n/a	>10
	Lactat dehydrogenase (LDH), U/L	847	n/a	n/a	n/a	140-280

Urine analysis	WBC: 1, red blood corpuscles: 0, no bacteria.					

n/a: not available.

## Data Availability

The dataset used and/or analyzed during the current study are available from the corresponding author on reasonable request.
